# Supplementary material for: Factors affecting early mobilization among critically Ill patients in Southern West Bank Hospitals
Source: PLoS One. 2025 Jun 25;20(6):e0325457. doi: 10.1371/journal.pone.0325457 (PMC12194204; doi:10.1371/journal.pone.0325457)
Supplement: S1 File — The validated tool used to assess patient mobility levels and barriers in the ICU. (DOCX) [file pone.0325457.s001.docx]

**S1 File. PERME ICU Mobility Tool**

PAGE 1

| Tools | Patient name of number | Date Time |
| --- | --- | --- |
| Mental Status Maximum points=3 | 1. Alertness upon arrival Unresponsive=0 Lethargic=1  Awake and alert=2 |  |
|  | 2. Is the patient able to follow 2 out of 3 commands |  |
| Potential Mobility Barriers  Maximum points=4  * Upon initial contact with the patient  or at any time during the mobility interventions | 1. Is the patient on mechanical ventilation or noninvasive? *   Yes=0 No=1   1. Pain *   Unable to determine or patient indicates to be pain=0 No pain=1   1. The patient has 2 or more of the following   Supplemental oxygen device. Foley catheter, ETT, trach, central line, peripheral IV, arterial line, dialysis catheter, PICC, PEG, PEJ, nasogastric tube, chest tube, temporary pacemaker, pulmonary artery catheter, epidural PCA, IABP, LVAD, CRRT, ventriculostomy, lumbar drain, wound VAC, or other.  Yes=0 No=1 |  |
|  | 6. Is this the patient on any drips?* (continuous intravenously infusion: vasopressors, inotropes, insulin, antiarrhythmie, sedation, antibiotics, fluids, electrolyte replacement, blood transfusions, etc.)  Yes=0 No=1 |  |
| Functional Strength Maximum points=4 | 7. Legs: Is the patient able to raise the leg against gravity approximately 20 degrees, with knee straight?  Yes=0 No=1 |  |
|  | 8. Legs: Is the patient able to raise the arm against gravity approximately 45 degrees, with elbow straight?  Yes=0 No=1 |  |

PAGE 2

| Tools | Patient name of number | Date Time |
| --- | --- | --- |
| Bed Mobility  Maximum points=6 | 9. Supine to sit  Not assessed or total assistance (<25%)=0 Maximum assistance (25% to 50%)=1  Moderate assistance (50% to 75%)=2 Minimum assistance (>75%) or supervision=3  10. Static sitting balance on side of bed once position is established  Not assessed or total assistance (<25%)=0 Maximum assistance (25% to 50%)=1  Moderate assistance (50% to 75%)=2 Minimum assistance (>75%) or supervision=3 |  |
| Transfers  Maximum points=9 | 9. Sit to stand  Not assessed or total assistance (<25%)=0 Maximum assistance (25% to 50%)=1  Moderate assistance (50% to 75%)=2 Minimum assistance (>75%) or supervision=3  12. Static standing balance once standing position is established  Not assessed or total assistance (<25%)=0 Maximum assistance (25% to 50%)=1  Moderate assistance (50% to 75%)=2 Minimum assistance (>75%) or supervision=3  13. Transfer from bed to chair or chair to bed Not assessed or total assistance (<25%)=0 Maximum assistance (25% to 50%)=1  Moderate assistance (50% to 75%)=2 Minimum assistance (>75%) or supervision=3 |  |
| Gait  Maximum points=3 | 14. Gait  Not assessed or total assistance (<25%)=0 Maximum assistance (25% to 50%)=1  Moderate assistance (50% to 75%)=2 Minimum assistance (>75%) or supervision=3 |  |
| Endurance  Maximum points=3 | 15. Endurance:  Distance walked in 2 minutes, regardless of level of assistance required including rest periods (sitting or standing), with or without an assistive device.  Unable to walk or not assessed=0 Distance 5-50 feet=1  Distance 51-99 feet=2 Distance ≥ 100 feet=3 |  |
| Maximum points=32 | Total Points |  |
