# Supplementary material for: Factors affecting early mobilization among critically Ill patients in Southern West Bank Hospitals
Source: PLoS One. 2025 Jun 25;20(6):e0325457. doi: 10.1371/journal.pone.0325457 (PMC12194204; doi:10.1371/journal.pone.0325457)
Supplement: S2 Text — Description of statistical methods, software, and sample size justification. (DOCX) [file pone.0325457.s002.docx]

**S2 Text. Statistical Analysis Protocol**

- Software: IBM SPSS Statistics Version 27

- Analyses:

- Descriptive statistics (mean ± SD, frequencies).

- Independent t-tests and one-way ANOVA.

- Significance threshold: p< 0.05.

- Sample Size Justification: 95% confidence level, 5% margin of error.
