# Supplementary material for: Factors affecting early mobilization among critically Ill patients in Southern West Bank Hospitals
Source: PLoS One. 2025 Jun 25;20(6):e0325457. doi: 10.1371/journal.pone.0325457 (PMC12194204; doi:10.1371/journal.pone.0325457)
Supplement: Attachment — Submitted filename: Response to Reviewers.docx [file pone.0325457.s004.docx]

**Point-by-Point Response Table**

| **Reviewer/Editor** | **Comment Number** | **Item** | **Comment** | **Response** |
| --- | --- | --- | --- | --- |
| **Editor** | **1** | **Style Requirements** | Ensure manuscript meets PLOS ONE’s formatting guidelines. | Thank you for your guidance. The manuscript has been reformatted using PLOS ONE templates, including title page structure, section headings, and reference style. |
| **Editor** | **2** | **Ethics Statement** | Provide details on consent type (written/verbal) and IRB approval. | Thank you for highlighting this. The ethics statement in the Methods section now specifies: *“Written informed consent was obtained from awake patients or legally authorized representatives. Ethical approval was granted by the IRB of Palestine Ahliya University (Approval Code: CAMS/CCNA/26/524).”* |
| **Editor** | **3** | **Non-Blinded Review** | Include all author details removed during initial submission. | Thank you. All author names, affiliations, and contact details are now fully visible in the manuscript. |
| **Editor** | **4** | **Data Availability** | Confirm raw data accessibility or restrictions. | Thank you. The Data Availability Statement (post-Acknowledgments) clarifies: *“De-identified data are available on request from the corresponding author due to ethical restrictions. The PERME tool is included as S1 File.”* |
| **Editor** | **5** | **Supporting Information** | Add captions for Supporting Information files. | Thank you. A “Supporting Information” section has been added post-References, listing S1 File (PERME tool), S2 Text (ethics), and S3 Text (statistical protocol). |
| **Reviewer #1** | **1** | **Abstract** | Replace “Discussion” with “Conclusion”; remove unsupported claims. | Thank you for this critical observation. The Abstract now uses “Conclusion” and focuses only on findings directly reported in Results (e.g., pain, SD=0.0 explanation). |
| **Reviewer #1** | **2** | **Literature Review** | Integrate into Introduction; reduce redundancy. | Thank you. The standalone “Literature Review” section was removed. Key studies are now integrated into the Introduction (pp. 2–3), with reduced redundancy. |
| **Reviewer #1** | **3** | **Population** | Clarify inclusion of ICU vs. non-ICU patients. | Thank you. The Methods section now specifies: *“Participants were sampled exclusively from ICUs in six hospitals. Medical wards and rehab facilities were excluded.”* |
| **Reviewer #1** | **4** | **Translation Process** | Detail cultural adaptation steps. | Thank you. The Methods section now describes: *“Forward-backward translation by bilingual experts and pilot testing with ICU nurses/physicians.”* |
| **Reviewer #1** | **5** | **Statistical Software** | Report software used. | Thank you. The Methods section now states: *“Analyses were performed using IBM SPSS v28.”* |
| **Reviewer #2** | **1** | **Missing References** | Add citations for EM barriers in resource-limited settings. | Thank you. Citations for regional challenges (e.g., [6,7]) were added to the Introduction. |
| **Reviewer #2** | **2** | **SD=0.0 Anomaly** | Justify homogeneity in abdominal pain subgroup. | Thank you. The Results and Discussion now clarify: *“SD=0.0 reflects identical PERME scores in abdominal pain patients (n=42), likely due to minimal barriers.”* |
| **Reviewer #2** | **3** | **Pain Management** | Recommend actionable strategies. | Thank you. The Discussion now proposes: *“Pre-activity analgesia and CPOT assessments to reduce pain-related reluctance.”* |
| **Reviewer #2** | **4** | **Exclusion Rationale** | Explain the exclusion of patients with disabilities. | Thank you. The Limitations section states: *“Excluding pre-existing disabilities limits insights into tailored interventions for high-risk groups.”* |
